# Health Information Technologies in the Support Systems of Pregnant Women and Their Caregivers: Mixed-Methods Study

**DOI:** 10.2196/10865

**Published:** 2019-05-09

**Authors:** Marian Taylor Dorst, Shilo H Anders, Sai Chennupati, Qingxia Chen, Gretchen Purcell Jackson

**Affiliations:** 1 Vanderbilt University Nashville, TN United States; 2 Vanderbilt University Medical Center Nashville, TN United States

**Keywords:** pregnancy, social networks, social media, health information technology, caregivers, life stress

## Abstract

**Background:**

The quality and quantity of families’ support systems during pregnancy can affect maternal and fetal outcomes. The support systems of expecting families can include many elements, such as family members, friends, and work or community groups. Emerging health information technologies (eg, social media, internet websites, and mobile apps) provide new resources for pregnant families to augment their support systems and to fill information gaps.

**Objective:**

This study sought to determine the number and nature of the components of the support systems of pregnant women and their caregivers (eg, family members) and the role of health information technologies in these support systems. We examined the differences between pregnant women’s support systems and those of their caregivers and the associations between support system composition and stress levels.

**Methods:**

We enrolled pregnant women and caregivers from advanced maternal-fetal and group prenatal care clinics. Participants completed surveys assessing sociodemographic characteristics, health literacy, numeracy, and stress levels and were asked to draw a picture of their support system. Support system elements were extracted from drawings, categorized by type (ie, individual persons, groups, technologies, and other) and summarized for pregnant women and caregivers. Participant characteristics and support system elements were compared using the Pearson chi-square test for categorical variables and Wilcoxon ranked sum test for continuous variables. Associations between support system characteristics and stress levels were measured with Spearman correlation coefficient.

**Results:**

The study enrolled 100 participants: 71 pregnant women and 29 caregivers. The support systems of pregnant women were significantly larger than those of caregivers—an average of 7.4 components for pregnant women and 5.4 components for caregivers (*P*=.003). For all participants, the most commonly reported support system elements were individual persons (408/680, 60.0%), followed by people groups (132/680, 19.4%), technologies (112/680, 16.5%), and other resources (28/680, 4.1%). Pregnant women’s and caregivers’ technology preferences within their support systems differed—pregnant women more often identified informational websites, apps, and social media as parts of their support systems, whereas caregivers more frequently reported general internet search engines. The size and components of these support systems were not associated with levels of stress.

**Conclusions:**

This study is one of the first demonstrating that technologies comprise a substantial portion of the support systems of pregnant women and their caregivers. Pregnant women more frequently reported specific medical information websites as part of their support system, whereas caregivers more often reported general internet search engines. Although social support is important for maternal and fetal health outcomes, no associations among stress, support system size, and support system components were found in this study. As health information technologies continue to evolve and their adoption increases, their role in patient and caregiver support systems and their effects should be further explored.

## Introduction

### Background

Significant and meaningful social support has been generally accepted as a means of positively affecting a pregnant mother’s well-being. Specifically, it mitigates her stress, improves her eventual birth outcomes, and buffers against prenatal and postpartum depression [1-5]. Social support also has functional value as members of the support system can provide material and physical assistance as well as emotional and informational feedback [6,7]. Although some literature has suggested that the amount and quality of social support does not improve a pregnant mother’s stress coping mechanisms or birth outcomes, a positive association between social support and mother’s and child’s well-being has prevailed in many studies [8,9]. There are many types of social support that contribute to a pregnant mother’s well-being. Social relationships describe an individual’s more immediate social ties, including family members, marriage partners, and close friends [10]; social resources are those relationships that are identified by an individual as helpful [11]. This important distinction differentiates between a pregnant mother’s social connections and the subset of these personal connections whose support she finds most valuable and the least stress inducing. The combination of a pregnant woman’s social relationships and social resources creates her unique social support system that helps her navigate pregnancy [12].

Not only is the physical presence of a social support system important but also is the perceived support of this system. Perceived social support has been shown to have a positive effect on stress levels and quality of life [13,14]. Specifically for pregnant women, perceived social support has been shown to be increased when using health information technologies such as online blogs and Facebook [13,15]. This greater perceived social support leads to higher perceptions of individual empowerment and improved maternal well-being, especially when looking at the variables of stress, depression, and marital satisfaction and conflict [16,17].

To build a strong and well-perceived support system, pregnant women may employ the help of caregivers to assist with their emotional and physical needs during this life stage [14,18]. In the context of this study, a caregiver is any informal, that is, any nonprofessional assistant who provides care for the pregnant woman [19,20]. These caregivers could include individuals such as spouses, parents, or friends, and they are an invaluable source of support [18,20]. However, it is important to recognize that providing this support can cause stress for the caregivers themselves. Previous research has shown that caregivers can experience negative health outcomes and psychological distress while fulfilling the caregiver role [20]. Therefore, it is necessary to investigate the support systems of both pregnant women and their caregivers as both these groups may experience stress during the pregnancy.

As consumer health information technologies have proliferated and evolved, they have also become popular resources for pregnancy support. According to a recent study of 613 women from throughout the world, 97% of pregnant mothers with internet access used general search engines to seek pregnancy-related information for a variety of reasons, including unsatisfactory experiences with health care providers, social networking, and general pregnancy information [21]. Women’s confidence in making pregnancy-related decisions can increase significantly after consulting internet sources. Researchers have observed that smartphone apps and social media platforms combine the “expert patient” ideal, the desire to feel up to date with all available health information on the internet, with the ideologies of responsible motherhood [22]. Many of these apps are considered useful by mothers because they *push* content. Instead of mothers having to seek out relevant information, pregnancy apps often send regular emails and updates to their users about temporal fetal development and the stages of pregnancy. There have also been recent albeit limited efforts to create health-related technologies for expectant fathers and other caregivers of the pregnant mothers. For example, an Australian app, mPregnancy, designed for men, includes more masculine-oriented descriptions of the phases of fetal development and stages of pregnancy [22].

### Goal of This Study

Little has been published about the presence and role of health information technologies in the support networks of pregnant women and their caregivers. As part of a comprehensive study of health information needs and information management practices in pregnant families, we aimed to elicit the components of the social networks of pregnant women and their caregivers, the types of technologies considered part of this support system, and any relationships between social network characteristics and stress. With these data, this study sought to identify the prevalence of technology in the social networks of pregnant women and their caregivers to expose a potential need for increased and improved technological resources for this population.

## Methods

### Study Population

We enrolled 100 pregnant women and their caregivers from 2 diverse prenatal care settings at the Vanderbilt University Medical Center (VUMC): the Junior League Fetal Center at Vanderbilt (FCV) and the Expect with Me (EWM) group prenatal care program. The FCV is an advanced maternal-fetal care setting that incorporates a clinical program in fetal diagnosis and therapy. Multidisciplinary teams bring a group of expert medical providers from different specialties to deliver care at 1 location. Most patients seen at the FCV experience a pregnancy with a fetal anomaly or other complications of pregnancy. EWM is an innovative group prenatal care program that combines the components of traditional prenatal care with health education and support delivered in a group setting. Small groups of pregnant women (ie, 8-12 individuals) with similar gestational ages and their caregivers meet for 10 group sessions during pregnancy. This model is aligned with the Institute of Medicine’s six domains of quality and has been demonstrated to reduce preterm births and health care costs during and after birth [23]. Inclusion of these 2 settings ensured that this study included both normal pregnancies and pregnancies with complications.

Adult pregnant women and then their caregivers were approached at both sites for participation in the study; if interested, they contacted us to set up an interview time. Pregnant women were approached for participation first. If she agreed to participate, she could then choose whether or not to include up to 3 caregivers in the study as well. Not all pregnant women chose to include caregivers in the study; nor did all pregnant women have caregivers available for participation. The eligibility criteria for all participants included age greater than or equal to 18 years, pregnancy with gestational age less than 36 weeks, home within 100 miles of VUMC, and the ability to speak English or Spanish. All participants provided written informed consent. This study was approved by the VUMC institutional review board.

### Measures

This research project contains analyses of data from a comprehensive study of information needs and information management practices in pregnant women and their caregivers. All participants completed an individual research visit to VUMC during which they completed surveys and participated in an interview, all of which typically lasted between 1 and 2 hours. For Spanish-speaking participants, all research materials were translated using a forward and backward translation process into Spanish, and a Spanish interpreter was present for the entire research visit. Participants were compensated for their time with a US $25 gift card and reimbursement for their travel to and from VUMC. The measures relevant to this specific research project are described below.

The participants completed a demographic questionnaire assessing age, race, ethnicity, home location, marital status, parity (number of children previously born), employment status, individual and household income, education, and access to various technologies. We recorded the gestational age of the pregnancy at the date of the interview and the relationship between the pregnant woman and any participating caregivers. Additional surveys were used to assess health literacy, numeracy, and levels of stress. For English-speaking participants, health literacy was measured using the Rapid Estimate of Adult Literacy in Medicine (REALM), a well-validated and widely used measure of health literacy [24,25]. For Spanish-speaking participants, health literacy was assessed with the Short Assessment of Health Literacy for Spanish-speaking Adults, which is based on and highly correlated with REALM [26]. Numeracy was measured by the General Health Numeracy Test, a 6-item validated questionnaire for assessing general health numeracy [27]. Stress was determined using the Cohen Perceived Stress Scale, a widely used psychological instrument for measuring the perception of stress that has demonstrated high reliability in pregnant populations [28,29] and has been translated into Spanish with adequate fit [30,31]. This instrument is a questionnaire in which various feelings are assessed on a 5-point scale, ranging from never (0) to almost always (4). Positively worded items are reverse scored, and the ratings are averaged, such that a higher score indicates more perceived stress. We examined stress levels because in the pregnant population, high stress levels have been associated with decreased birth weight of babies as well as preterm delivery [32] and because of reported associations between social networks and stress during pregnancy. Studies involving other health-related diagnoses have found correlations between meeting information needs and reductions in stress [33].

Support resources were assessed by giving participants a sheet of paper with a face in the center, representing the participant, and the following instructions:

Draw or list your support system and indicate the key people/sources that you have relied on as resources during this pregnancy. The most important people should be closer to you or indicated with an *. Also, tell us who these people are. For example, they might be a member of your family, friend, healthcare professional, or internet resource (e.g., contacts from blogs, social networking sites, or other web or mobile application resources).

This method for gathering this information is in line with participatory design methods and has been used successfully in other studies [34]. The interviewer would read the instructions and answer any questions that the participant had about this measure.

**Figure 1:**
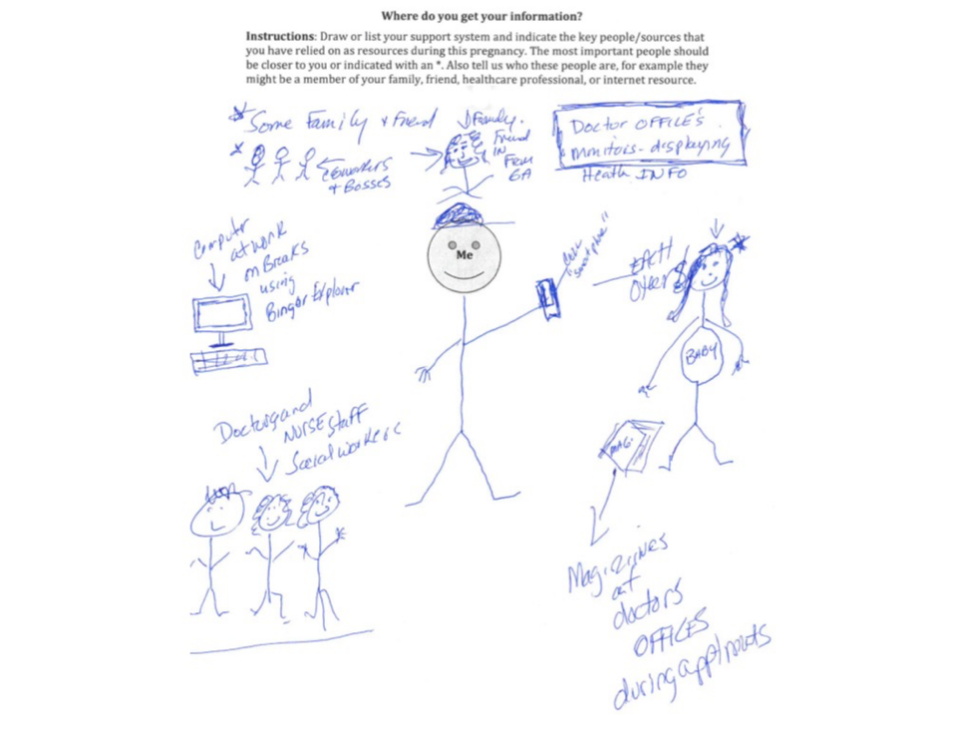
Example of caregiver support network.

An example of a caregivers’ support network is shown in Figure 1.

### Analyses

From each social resource drawing, research team members extracted the total number of elements and determined the number of individual people, groups of people, technologies, and other elements. The independent variable we noted was the role of each person (eg, husband, sister, or mother), and the dependent variables were the name or type of each group and the types of technologies. Subtypes of technologies were categorized as general internet search engines (eg, Google), educational or informational resources (eg, WebMD), apps (eg, BabyBump), or social media (eg, Facebook).

For continuous characteristics, we summarized the data using mean and SD, and we compared these variables between pregnant women and caregivers with the use of the Wilcoxon rank sum test. For categorical characteristics, we reported the percentages of each category and compared between roles using the Pearson chi-square test. Stress level was evaluated by 10 items, with each given a value ranging from 0 to 4. The internal consistency of items was measured by the Cronbach coefficient. With good to excellent internal consistency (Cronbach alpha >.8), the stress score would be calculated as the average of nonmissing 10-item values. Associations among support system size, components, and stress were examined with the Spearman correlation for continuous variables and Pearson chi-square test for categorical variables. The 95% CI was constructed for Spearman correlation coefficient based on 5000 bootstrap samples. The 2-sided *P* values less than .05 were considered significant. All analyses were performed using R3.4.4 (the R Project for Statistical Computing). These analyses were conducted using the lens of grounded theory: empirical data are coded, categorized, and interpreted to recognize patterns and theories [35].

## Results

### Population

This study enrolled a total of 100 participants, including 71 pregnant women and 29 caregivers. For the 29 caregivers, the relationships with the mother consisted of 11 spouses, 7 significant others, 7 parents, 2 siblings, 1 child older than 18 years, and 1 ex-husband. The characteristics of the study population are shown in Table 1. Overall, 83 participants were recruited from the FCV and 17 from EWM. In addition, 79.0% (79/100) of our sample was female and 21.0% (21/100) male. Pregnant participants had an average age of 27.6 years (SD 6.3 years), whereas caregivers were significantly older, with an average age of 33.9 years (SD 12.0 years; *P*=.04). Furthermore, 20.0% (20/100) of participants identified as black or African American, 71.0% (71/100) as white, 1.0% (1/100) as Native Hawaiian or other Pacific Islander, and 8.0% (8/100) as other. In addition, 5 participants (4 pregnant women and 1 caregiver) were Spanish speaking.

**Table 1:** Demographic characteristics of the study population.

Demographic characteristic		Pregnant women (n=71)	Caregivers (n=29)	Total (N=100)
Age, mean (SD)		27.6 (6.3)	33.9 (12.0)	29.4 (8.8)
Estimated gestational age, mean (SD)		30.8 (4.0)	31.1 (3.9)	30.9 (4.0)
**Race, n (%)**				
	American Indian or Alaska Native	0 (0)	0 (0)	0 (0)
	Asian	0 (0)	0 (0)	0 (0)
	Black or African American	14 (20)	6 (21)	20 (20)
	Native Hawaiian or other Pacific Islander	1 (1)	0 (0)	1 (1)
	White	50 (70)	21 (72)	71 (71)
	Other	6 (8)	2 (7)	8 (8)
**Gender, n (%)**				
	Female	71 (100)	8 (28)	79 (79)
	Male	0 (0)	21 (72)	21 (21)
**Other children, n (%)**				
	Yes	36 (51)	17 (59)	53 (53)
	No	35 (49)	12 (41)	47 (47)
**Education level**, **n (%)**				
	Eighth grade	0 (0)	1 (3)	1 (1)
	High school	22 (31)	11 (38)	34 (34)
	Some college courses	17 (24)	8 (28)	25 (25)
	2-year degree	6 (8)	2 (7)	8 (8)
	4-year degree	14 (20)	5 (18)	19 (19)
	Master’s degree	7 (10)	1 (3)	8 (8)
	PhD or equivalent	2 (3)	0 (0)	2 (2)
	Other	3 (4)	1 (3)	3 (3)
**Individual income, n (%)**				
	Under US $15,000	34 (51)	7 (26)	41 (41)
	US $15,000-US $29,999	12 (18)	9 (33)	21 (21)
	US $30,000-US $44,999	8 (12)	6 (22)	14 (14)
	US $45,000-US $59,999	4 (6)	2 (7)	6 (6)
	US $60,000-US $79,999	5 (7)	1 (4)	6 (6)
	US $80,000-US $99,999	2 (3)	1 (4)	3 (3)
	US $100,000 and above	2 (3)	1 (4)	3 (3)
**Household income,** **n (%)**				
	Under US $15,000	13 (19)	6 (22)	19 (19)
	US $15,000-US $29,999	13 (19)	7 (26)	20 (20)
	US $30,000-US $44,999	11 (16)	7 (26)	18 (18)
	US $45,000-US $59,999	9 (13)	4 (15)	13 (13)
	US $60,000-US $79,999	5 (8)	0 (0)	5 (5)
	US $80,000-US $99,999	4 (6)	0 (0)	4 (4)
	US $100,000-US $119,000	5 (8)	0 (0)	5 (5)
	US $120,000-US $139,000	2 (3)	2 (7)	4 (4)
	US $140,000 and above	5 (8)	1 (4)	6 (6)
Stress, mean (SD)		1.4 (0.6)	1.7 (0.7)	1.6 (0.7)
Health literacy, mean (SD)		62.6 (5.2)	61.1 (6.4)	62.2 (5.6)

The average gestational age of the fetus at the time participants were interviewed was 30.9 weeks, 30.8 weeks for pregnant participants, and the pregnant woman associated with the caregiver was 31.1 weeks’ average gestational age at the time of their caregiver’s interview. Overall, 53.0% (53/100) of participants had children before the pregnancy looked at in this study. Health literacy scores (calculated out of 66 maximum points) averaged 62.2 for all study participants, 62.6 for pregnant women, and 61.1 for caregivers. A score of 61 or greater correlates to a ninth-grade literacy level. Individuals at this literacy level will be able to read most health education materials [24,25]. Health numeracy scores (calculated out of 6 maximum points) averaged 2.2 for all participants, 2.1 for pregnant women, and 2.3 for caregivers. Stress levels (range 0-4) averaged 1.6 for all participants, 1.4 for pregnant women, and 1.7 for caregivers. Between pregnant women and their caregivers, there were no significant differences in any of the variables (age, race, gender, etc) shown in Table 1 (range of *P*=.06 for stress levels between the groups to *P*=.92 for difference in race between the 2 groups).

### Technology Access

Table 2 presents the technologies that pregnant women and their caregivers reported having access to at the time of their participation. The most participants in both groups reported having smartphones, including 96% (68/71) of pregnant women and 93% (27/29) of caregivers. In both groups, fewer participants had home phones, specifically 37% (26/71) of pregnant women and 41% (12/29) of caregivers. Within the caregiver subset, there was a difference in access to technologies based on the caregiver’s sex. Overall, female caregivers reported lower rates of technology access across all categories. Female caregivers had the least access to home phones and gaming consoles, with 25% (2/8) reporting access in both categories, whereas male caregivers had the least access to just home phones (10/21, 48%).

### Support System Components

Tables 3 and 4 present the types and numbers of support system components for pregnant women and their caregivers. The 100 participants reported a total of 680 support system components. Individual persons were the most common support system elements, comprising 60.3% (408/680) of the support system for all participants, 58.4% (307/525) for pregnant women, and 57.7% (90/156) for all caregivers. Among the individuals cited, first-degree relatives such as spouses, parents, or siblings were the most common, although many individuals also included specific health care providers or named friends. The next most common support component was groups of people, making up 19.3% (132/680) of the support system for the entire sample, 21.0% (110/525) for pregnant women, and 21.2% (35/156) for caregivers. Friends, especially those with children, were one of the most common groups in support networks. Other commonly mentioned groups included colleagues, health care practices, and members of one’s church.

Both groups identified technologies as parts of their support system: 16.2% (112/680) for all participants, 16.8% (88/525) for pregnant women, and 16.0% (25/156) for caregivers. Google (20/112, 17.9% technology components), internet searches (21/112, 18.8%), and BabyCenter (14/112, 12.5%) were the most common support network components, but a wide variety of health information technologies were identified, including blogs, online support groups, and online journals. Finally, other resources comprised the smallest part of the support system: 4.2% (28/680) of the system for all participants, 3.8% (20/525) for pregnant women, and 5.1% (8/156) for caregivers. In this category, magazines and books, most notably the book *What to Expect When You’re Expecting*, were the most common elements.

**Table 2:** Pregnant women’s and caregivers’ current access to technologies.

Technology	All participants (N=100), n (%)	Pregnant Women (n=71), n (%)	Caregivers (n=29), n (%)	Female caregivers (n=8), n (%)	Male caregivers (n=21), n (%)
Computer at home	81 (81)	59 (83)	22 (76)	5 (63)	17 (81)
Computer at work	63 (63)	42 (59)	21 (72)	3 (38)	18 (86)
Smartphone	95 (95)	68 (93)	27 (93)	7 (89)	20 (95)
Cell phone	92 (92)	66 (93)	26 (90)	6 (75)	20 (95)
Home phone	38 (38)	26 (37)	12 (41)	2 (25)	18 (86)
Gaming console	66 (66)	46 (65)	20 (69)	2 (25)	10 (48)
Tablet	68 (68)	49 (69)	19 (66)	3 (38)	16 (76)

**Table 3:** Types of support system components.

Participants		Support system components										
		Persons		Groups		Technology		Others			Totals	
		Total, n (%)	Mean (SD)	Total, n (%)	Mean (SD)	Total, n (%)	Mean (SD)	Total, n (%)	Mean (SD)	Total, n		Mean (SD)
All participants (N=100)		408 (60.0)	3.97 (2.98)	132 (19.4)	1.45 (1.48)	112 (16.5)	1.13 (1.19)	28 (4.1)	0.28 (0.51)	680		6.8 (3.4)
Pregnant women (n=71)		307 (58.4)	4.32 (3.32)	110 (21.0)	1.55 (1.50)	88 (16.8)	1.24 (1.21)	20 (3.8)	0.28 (0.54)	525		7.4 (3.40)
**Caregivers (n=29)**		90 (57.7)	3.10 (1.80)	35 (22.4)	1.13 (1.38)	25 (16.0)	0.86 (1.13)	8 (5.1)	0.28 (0.45)	156		5.4 (3.0)
	Female (n=8)	16 (57.1)	2.00 (0.93)	9 (32.1)	1.13 (0.32)	1 (3.6)	0.13 (0.35)	2 (7.1)	0.25 (0.46)	28		3.5 (2.0)
	Male (n=21)	74 (57.8)	3.52 (1.89)	24 (18.8)	1.14 (1.20)	24 (18.8)	1.14 (1.20)	6 (4.7)	0.29 (0.46)	128		6.1 (3.0)

**Table 4:** Technology support system components.

Participants		Technology support system components				
		General internet search engines, n (%)	Informational or educational websites, n (%)	Apps, n (%)	Social media tools, n (%)	Total, n (%)
All participants (N=100)		40 (37.3)	45 (42.1)	10 (9.3)	12 (11.2)	107 (100)
Pregnant women (n=71)		29 (33.7)	36 (41.9)	10 (11.6)	11 (12.8)	86 (100)
**Caregivers (n=29)**		11 (50)	9 (40.9)	0 (0)	2 (9.1)	22 (100)
	Female caregivers (n=8)	1 (100)	0 (0)	0 (0)	0 (0)	1 (100)
	Male caregivers (n=21)	10 (47.6)	9 (42.9)	0 (0)	2 (9.5)	21 (100)

When the caregiver subset was divided into men and women, there were some apparent differences in the support system composition. As seen in Table 3, male and female caregivers showed a similar proportion of persons in their support system but differed in their proportions of groups, technology, and other elements. Female caregivers’ support systems included a greater percentage of groups (9/28, 32.1%) than did the support systems of males (24/128, 18.8%), and men reported a greater proportion of technology in their support systems (24/128, 18.8%) than did women (1/28, 3.6%). Men also showed a greater mean number of support elements in every category. Pregnant women had slightly higher mean number of components, mainly from persons and technology. For caregivers, the most notable differences between the genders were within the categories of persons and technology. Male caregivers’ support systems, on average, included 3.52 support persons and 1.14 technology elements, whereas female caregivers’ support systems consisted of 2.00 persons and 0.13 technology elements.

### Associations With Stress

We examined whether the participant characteristics, including the social network size and components, were associated with levels of stress. Stress was not found to significantly correlate with age (Spearman rho=−0.176; 95% CI −0.361 to 0.021; *P*=.08), race (*P*=.93), household income (Spearman rho=−0.196; 95% CI −0.395 to 0.008; *P*=.06), or education level (Spearman rho=−0.111; 95% CI −0.286 to 0.07; *P*=.27) within the entire sample. None of these variables within the pregnant women and caregiver data subsets significantly correlated with stress (all *P*>.05). In addition, none of the support system components described in Table 3 significantly correlated with stress; among all participant data, pregnant women subset data, and caregiver subset data, there was no significant correlation between stress and the number of persons, groups, technology, and other resources in an individual’s support system. The composition of an individual’s support system (ie, percentage of support system distributed across persons, groups, technology, and other support components) also did not correlate significantly with stress. Only 1 variable, individual income, correlated with stress level (Spearman rho=−0.229; 95% CI −0.425 to −0.026; *P*=.03).

### Technology Support System Components

Table 4 presents the types of technologies pregnant women and their caregivers reported as part of their support systems. Among all the participants, informational websites were the most popular form of technology support resources (45/107, 42.1%), followed by general internet search engines (40/107, 37.3%), social media tools (12/107, 11.2%), and apps (10/107, 9.3%). Among pregnant women, informational websites are the most popular form of technological support (36/86, 41.9%), followed by general internet search engines (29/86, 33.7%), social media tools (11/86, 12.8%), and apps (10/86, 11.6%). Caregivers more frequently reported general internet search engines (11/22, 50%) as part of their support systems, followed by informational websites (9/22, 40.9%), social media tools (2/22, 9.1%), and then apps (0/22, 0%). Consistent with Table 2, only 1 female caregiver reported a technology support element (general internet search engine), whereas male caregivers reported support using general internet search engines (10/21, 47.6%), informational websites (9/21, 42.9%), and social media tools (2/21, 9.5%).

## Discussion

### Principal Findings

This study is one of the first to examine the presence of health information technologies as part of the support systems of pregnant women and their caregivers [15-17,22]. We observed that both pregnant women and a diverse set of caregivers reported technologies as a substantial component of the support systems for pregnancy. Pregnant women more frequently cited medical informational sites rather than broad internet search engines as components of their support system, with social media and apps identified less frequently. Caregivers more often included general internet search engines in their support networks, followed by informational websites, social media, and apps. These differences suggest that pregnant women may have more specific questions regarding their own pregnancy and the medical conditions of both themselves and the fetus. Therefore, they seek more specific advice from reputable informational websites such as hospital or government sites rather than search broadly using sites such as Google. Previous research has suggested that prenatal visits are ineffective in addressing the information needs of pregnant women, and therefore, they may turn to technologies and online resources to fill these gaps [36].

We also noted that male caregivers reported more individual and technology support system components than female caregivers did. This finding may expose a current need for pregnancy-related technology and resources specifically designed for male caregivers as most pregnancy resources are likely tailored for expectant mothers and a female audience. Previous research has found that during pregnancy, male caregivers are often portrayed as the “bumbling assistants,” and thus, the resources available to them are written with oversimplified information [22]. In actuality, male caregivers may wish to play a more serious and informed role, and current technology resources do not support this role. Our analysis of information needs reported by participants in this study revealed that male caregivers do indeed want information about the phases of pregnancy, normal versus abnormal symptoms of a pregnant woman, and other relevant information to assist the pregnant individual [37]. However, currently available information may be written primarily with a pregnant audience in mind, which could limit the understanding of a male caregiver and reinforce the “bumbling assistant” stereotype [22]. Fathers often feel excluded from the prenatal education process [38], and our findings might suggest that our caregivers sought additional information and support through a greater number of persons and technologies in their network. Male caregivers also reported greater access to all forms of technology than did female caregivers, so their greater inclusion of technology-based resources as support system components may have been observed simply because of this greater access.

Overall, pregnant women had larger support systems than their caregivers, suggesting that expectant mothers seek larger and broader support networks during the vulnerable life stage of pregnancy. Pregnant women identified more persons, groups, and technologies as parts of their support systems than did caregivers. This finding supports the results of previous research, which demonstrated that first-time mothers reported more numerous sources of support than their husbands [38]. This may insinuate that a pregnant woman is offered more help during her pregnancy through a variety of personal and informational resources. Alternatively, she may simply be more receptive of the aid provided during this time in her life.

In our study, women also valued information and support from experienced mothers and women who had recently given birth. One pregnant participant in our sample reported that she regularly sought information on Facebook from her high school friends who lived in another state; they were also pregnant and having children, and they would provide information tailored to her. This finding supports previous research that pregnant women who invested time into online blogs and Facebook groups reported feeling more supported in their transition to motherhood and more connected to extended family, friends, and the outside world [15-17]. Technologies such as social media may provide a way for both pregnant women and caregivers to obtain support from family and friends who are geographically remote.

Female caregivers, such as paternal and maternal grandmothers of the child, have been shown to play a central role in pregnancy decision making and in postnatal decisions such as breastfeeding by sharing their experience [39]. Female caregivers such as grandmothers are often seen as possessors of time-tested knowledge, and thus, they are able to significantly influence a pregnant woman, both positively and negatively [39]. One caregiver in our study, who was a mother to the pregnant participant, reported using social media to share her knowledge and experience with expectant mothers within and outside her family. As older age groups continue to adopt and use social media, this type of support may become more commonplace. However, in our study, we see that both pregnant women and female caregivers reported people-based resources such as individuals and groups in their support systems much more frequently than technologies. Female caregivers may both contribute toward and benefit from prenatal group-style programs such as EWM where they can share, compare, and gain knowledge. The predominance of persons in the support networks of pregnant women may have been affected by recruitment from such a group prenatal care setting, giving these participants and their caregivers greater access to persons with shared experiences to build their support networks.

Both pregnant women and their caregivers also indicated that groups comprised a substantial portion of their support systems. Many cited groups in support networks were large, for example, a church congregation or a physician group/midwifery team. Previous studies have shown that church attendance and involvement in clubs during pregnancy were not associated with increased perceived social support [40,41]. The high prevalence of religious and health care groups observed in the support networks in our study may have been a reflection of cultural norms in the southeastern United States and the intensive care provided in both of our recruitment sites.

Pregnant women more frequently noted social media tools as part of their support networks. The social media category includes interactive information searching, such as online forums, blogs, Facebook, and Pinterest, which may appeal more to the pregnant woman who seeks a more tailored, immediate, or relatable response to a question. The role of social media in meeting pregnancy information needs and augmenting a pregnant woman’s support network has not been extensively explored, but the potential health benefits to extending social networks through technologies are numerous. It is well known that health behaviors and conditions such as smoking and obesity are spread through in-person social networks, but the exact nature of how such health states are propagated has yet to be elucidated fully [41,42]. Positive health-related behaviors, such as smoking cessation and happiness, have also been shown to spread through social networks [43,44]. Online social networks and social media tools, such as Facebook and Twitter, are communication tools by which social ties can be formed, strengthened, and maintained, which can increase perceived social support [13,14]. One could envision pregnant women and their caregivers obtaining social support for important perinatal activities such as exercise and breastfeeding through health information technologies. No caregivers acknowledged using any sort of app in their support system, and only 1 caregiver reported the use of 2 social media tools. This supports the notion that most pregnancy-related apps are more directly targeted toward pregnant women rather than toward their caregivers.

Our findings correspond with a fundamental tenet of social network theory: when an individual is exposed to components of her social network, she may adjust her behavior via mechanisms such as social pressure and information gathering [45]. Though our study was not designed to collect data within a social network theory framework, it is interesting to consider how the strength, quality, and variety of our participants’ support systems may influence information-seeking behaviors.

Unlike previous studies, we did not see any relationship between the size or nature of the support system and stress [13]. The generally large breadth of our pregnant women’s and caregivers’ support systems may have offered the emotional and informational support that could counter stress. Our only variable that did significantly correlate with stress, individual income, is an external variable that is not easily stabilized or impacted by a support system. In addition, this research project was a cross-sectional study that examined support networks and stress before delivery, and both may change significantly after the birth of the child. The stress levels observed in our population were relatively low, and it is also possible that our sample of 100 participants may not be large enough to observe such a correlation between support system resources and stress. Our ongoing research includes a similar longitudinal study, which will examine changes in support systems and stress levels at various points during pregnancy and after delivery.

### Limitations

Our study has several limitations. First, only English- and Spanish-speaking participants were included, and relatively few Spanish-speaking individuals participated. The demographics of our population reflected those of the state of Tennessee and nearby communities, and our findings may not generalize to other settings. The size and nature of support systems likely have cultural influences, and our study may have excluded other populations with different types of social networks. Our study instrument also provided participants with potential examples that may have impacted their responses when completing this sheet. In addition, our study was cross-sectional, and the varying gestational ages of the participants could have influenced the responses given. For example, a pregnant woman at a gestational age of 34 weeks with delivery in the near future would consider her midwife and doctor as valuable support resources, but a pregnant woman at a gestational age of only 18 weeks may be more concerned with day-to-day support provided by family and colleagues. Our research team is currently conducting a longitudinal study in which support systems, technology usage, and stress will be evaluated during the course of pregnancy and after delivery. Finally, this study did not look more deeply into the types of support provided by each component of the support network. Therefore, we could not assess the quality of any support resource or make direct conclusions about its usefulness or purpose during the pregnancy experience of each participant.

### Conclusions

Social support is critical for optimal maternal and fetal outcomes during pregnancy. This study is one of the first to demonstrate that technologies formed a substantial component of the support networks for pregnant women and their caregivers. Pregnant women more frequently cited specific medical informational or educational resources, whereas caregivers more commonly reported general internet search engines in their support networks. Male caregivers reported more access to technologies and identified them in their support networks more frequently than did female caregivers. Female caregivers’ support networks during pregnancy included more individuals and groups of persons. Additional research is needed to explore exactly how technologies provide support to families during pregnancy and the effects that use of health information technologies might have on pregnancy outcomes.

## Abbreviations

EWM: Expect with Me
FCV: Junior League Fetal Center at Vanderbilt
REALM: Rapid Estimate of Adult Literacy in Medicine
VUMC: Vanderbilt University Medical Center
